# Imaging antigen processing and presentation in cancer

**DOI:** 10.1093/immadv/ltaf002

**Published:** 2025-02-04

**Authors:** Doreen Lau, Tim Elliott

**Affiliations:** Centre for Inflammation Research and Translational Medicine, Department of Life Sciences, Division of Biosciences, Brunel University London, London, United Kingdom; Department of Radiology, School of Clinical Medicine, University of Cambridge, Cambridge, United Kingdom; Centre for Immuno-Oncology, Nuffield Department of Medicine, University of Oxford, Oxford, United Kingdom

**Keywords:** antigen processing, antigen presentation, immunotherapy, imaging technologies, biomarkers

## Abstract

**Introduction:**

Antigen processing and presentation are vital processes of the adaptive immunity. These processes involve a series of intracellular and extracellular events, including the enzymology within cells during antigen processing, the loading and presentation of antigenic peptides on major histocompatibility complexes, the recruitment of T cells, their interaction with antigen-presenting cells, and the expression of adhesion, co-stimulatory and co-inhibitory molecules at the T cell immunological synapse. These events collectively fine-tune and sustain antigen recognition and T cell function. Dysregulation of this machinery can profoundly impact the efficacy of cancer immunotherapy. Imaging technologies have emerged as powerful tools for elucidating the mechanisms underlying antigen processing and presentation. By providing complementary perspectives into the cellular and molecular interactions at play, imaging has significantly enhanced our understanding of these complex immunological events in cancer. Such insights can improve the monitoring of immunotherapy responses, facilitate the identification of effective treatments, and aid in predicting patient outcomes.

**Methods:**

This review explores the role of imaging in studying antigen processing and presentation in the context of cancer.

**Conclusion:**

It highlights key considerations for developing imaging tools and biomarkers to detect components of these pathways. Additionally, it examines the strengths and limitations of various imaging approaches and discusses their potential for clinical translation.

## Graphical Abstract

**Graphical Abstract:**
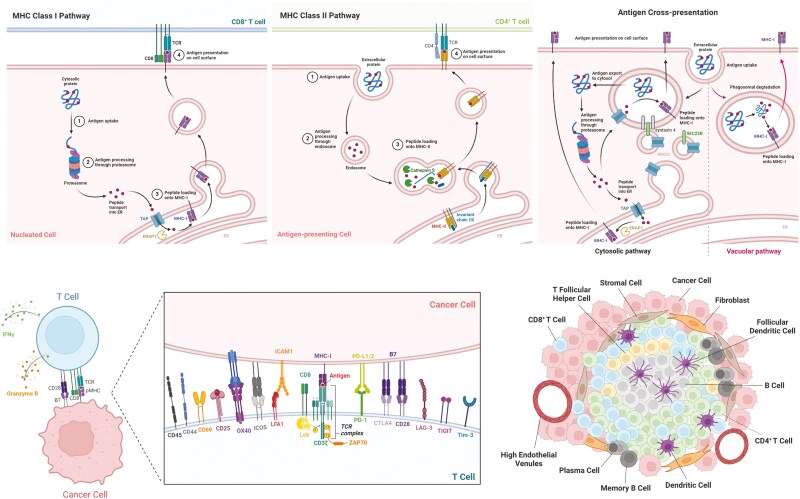

## Introduction

**Figure 1.**
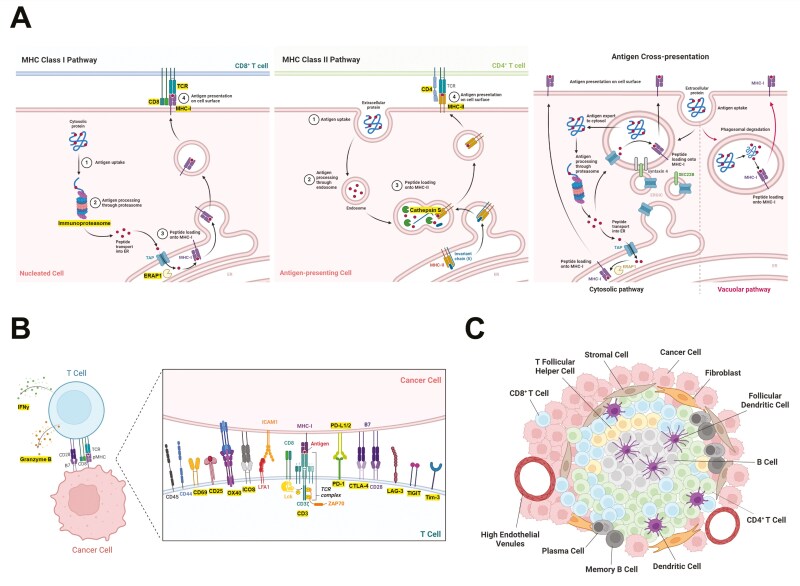
**Antigen processing and presentation in cancer.** (A) The Class I MHC pathway is initiated by the degradation of intracellular antigens into peptides by cytosolic and nuclear proteasomes in nucleated cells including cancer, followed by transportation of the peptides into the ER via TAP (transporter associated with antigen processing). ERAP1 further trims these peptides into appropriate length for loading onto MHC-I complexes. These peptide-MHC complexes are then transported to the cell surface for presentation to the TCR of CD8^+^ T cells. The Class II MHC pathway involves the internalization of exogenous antigens by APCs followed by antigen processing via the endocytic pathway. The complex of MHC-II and invariant chain (Ii) is transported through the Golgi into an acidic endo-lysosomal compartment where Cathepsin S digest Ii, leaving a residual class II-associated Ii peptide (CLIP) in the peptide-binding groove of MHC-II. Following the exchange of CLIP for an antigenic peptide, the peptide-MHC complex is transported to the cell surface for presentation to the TCR of CD4^+^ T helper cells. Professional APCs can process extracellular antigens into peptides via the cytosolic or vacuolar pathway for loading onto MHC-I complexes and presentation to CD8^+^ T cells in a specialized mechanism called cross-presentation. (B) CD8^+^ T cell activation and cytotoxic killing of cancer following antigen recognition is dependent on the quality of TCR signalling and the expression of co-stimulatory molecules, immune checkpoints, adhesion proteins, granzymes, cytokines etc. (C) Tertiary lymphoid structures are local hubs for antigen presentation, humoral response and adaptive immune activation in inflamed tissues such as cancer. They are made up of highly organized cellular clusters composed of T cells, APCs, high endothelial venules, stromal and fibroblast network. *Images created using BioRender.com (publication licence MY277PWG1D).* Key components of the antigen processing and presentation pathways that have been developed for imaging are highlighted in yellow.

Immunotherapy has revolutionized cancer treatment. Various interventions, such as immune checkpoint inhibitors, cancer vaccines, and engineered chimeric antigen receptor T cells, have been developed over the years to boost anti-tumour immunity [1]. Central to the effectiveness of these therapies is antigen processing and presentation (**Fig. 1**). Antigens are processed and presented to T cells by Class I or II major histocompatibility complex (MHC) molecules in the form of short peptides. Distinct intracellular pathways contribute to the processing and presentation of MHC-I and MHC-II restricted antigens: peptides are selected for presentation from a highly diverse pool of candidates—for MHC-I, these are mainly generated in the cytosol from defective ribosomal products and from the natural turnover of cellular proteins in a process known as *direct presentation*. For MHC-II, they are generated in intracellular vesicles resulting from the uptake of extracellular material, to which MHC-II molecules are specifically targeted and is generally called the *endosomal pathway* (even though other vesicles can be involved). In specialized antigen presentation, extracellular material can be delivered to the cytosol for processing and presentation by MHC-I in a process known as *cross-presentation* [2]. Any dysfunction in this machinery can significantly impair the immune system’s ability to recognize and respond to cancer cells, ultimately affecting treatment outcomes [3].

Understanding the complexities of tumour immunobiology during immunotherapy critically depends on advanced imaging techniques. Recent developments in molecular genetics, chemical biology, and imaging physics have enabled researchers to explore tumour spatial biology and the immune network with unprecedented detail, ranging from single molecules and cellular interactions to whole-body imaging. Imaging not only provides insights into cellular behaviours but also elucidates the molecular mechanisms underlying antigen processing and presentation.

In this review, we will examine the role of imaging in unravelling the complexities of antigen processing and presentation pathways as they operate *in vivo*. Key considerations for developing imaging tools and biomarkers that target various components of these pathways will be discussed. Additionally, we will assess the merits and limitations of these imaging approaches and explore the potential for clinical translation of some of these methods. This comprehensive overview will highlight how imaging can drive advancements in cancer immunotherapy, ultimately improving treatment monitoring, stratification and prediction of patient outcomes.

## Antigen processing

Antigen processing is a tightly regulated mechanism involving the intracellular degradation of cytosolic or exogenously derived antigens before peptide transportation to the right cellular compartments for association with MHC complexes. Several proteolytic enzymes participate in these processes and are associated with immunotherapy response. The opportunity to visualize and quantify changes in their levels of expression and function will be useful for disease diagnosis and treatment monitoring.

### Immunoproteasome

The immunoproteasome is a specialized type of protease complex that play key roles in the generation of antigenic peptides for Class I MHC presentation to CD8^+^ T cells. It is usually expressed constitutively in immune cells but can be induced in non-immune cells by interferon-gamma (IFNγ) or tumour necrosis factor-alpha (TNFα) during inflammation [4]. Immunoproteasome subtypes are defined by their subunits PSMB8 (LMP7), PSMB9 (LMP2) and PSMB10 (MECL-1) that are inducible by IFNγ stimulation. Overexpression of PSMB8 and PSMB9 are found to be predictive biomarkers of survival and improved response to immune checkpoint blockade in melanoma patients. Altered peptide repertoire and the production of more immunogenic peptides have also been found in cells overexpressing the immunoproteasome subunits [5]. Loss of immunoproteasome with epithelial-to-mesenchymal transition has been associated with poor prognosis in non-small cell lung carcinoma (NSCLC) [6]. Other than antigen processing, immunoproteasomes contribute to cytokine production and CD4^+^ T cell differentiation in the pathogenesis of autoimmune diseases. Thus, there is great pharmaceutical interest in developing immunoproteasome inhibitors, including the LMP7/2 inhibitor KZR-616, which is currently undergoing Phase II clinical trials in patients with lupus nephritis (*NCT05781750*).

Tissue penetrance, cell permeability, and specificity are some of the key factors to consider when developing imaging tools to detect the intracellular expression of immunoproteasomes. Activity-based probes (ABP) targeting the enzyme active site are typically used for monitoring proteasome activity in living cells. Carmony et al. have developed an ABP for imaging immunoproteasomes based on the structure of a LMP2 inhibitor (UK101) [7]. A linear hydrocarbon linker was introduced at the free amine functional group of UK101 to attach a fluorophore without affecting the probe interactions with LMP2. *In vitro* staining of PC-3 cells with different concentrations of a fluorescein labelled version of the probe (UK101-Fluor) showed that the probe binding to LMP2 was dose-dependent. Lower uptake of UK101-Fluor was detected in LMP2 knockdown cells compared to the control, indicating the probe specificity for LMP2. UK101-Fluor was showed to colocalize with calnexin staining in the ER. Specificity in detection of LMP2 was also demonstrated in a near-infrared (NIR) fluorescent modified version of the probe (UK101-B660).

Other than LMP2 imaging, a NIR probe (LKS01-B650) has been generated for visualizing the catalytically active LMP7 subunit of immunoproteasomes [8]. LKS01-B650 was based on the structure of an immunoproteasome inhibitor that selectively binds to LMP7 and was chemically modified with BODIPY 650/665 away from the interaction site with LMP7. Molecular dynamics simulation showed that LKS01-B650 retained good binding interactions with the X-ray crystal structure of LMP7 despite the addition of a fluorophore. *In vitro* staining revealed a reduction in BODIPY signal or competition in LKS01-B650 binding when Panc-1 cells were treated with a LMP7 inhibitor. LKS01-B650 was shown localizing to the perinuclear region where the assembled immunoproteasome complex is known to be situated. Interestingly, staining of Panc-1 cells with both LKS01-B650 and UK101-Fluor showed that the BODIPY signal indicating LMP7 expression was extensively co-localized with the fluorescein signals from LMP2 expression. These studies demonstrated feasibility in using ABPs for imaging the catalytic subunits of immunoproteasome. Furthermore, modification of immunoproteasome inhibitors with BODIPY dyes may enhance tissue penetration due to their lipophilic nature and facilitate NIR imaging on deep tissues for future *in vivo* studies. However, dose escalation and toxicology studies should be performed to ensure that the probes are only reporting on the enzyme activity but not causing unwanted therapeutic effects in the living system.

### Endoplasmic reticulum aminopeptidase 1

The endoplasmic reticulum aminopeptidase 1 (ERAP1) is important for antigen processing via the Class I MHC pathway. It resides in the ER lumen and regulates the peptide repertoire by trimming N-terminally extended peptides to an optimal length for loading onto MHC-I complexes [9]. Alterations in ERAP1 activity can influence the quality and quantity of peptides presented, thereby affecting T cell and natural killer (NK) cell responses. ERAP1 expression is ubiquitous in human tissues, with higher levels found in the heart, placenta and spleen. Similar to other members of the antigen processing and presentation pathway such as MHC-I and tapasin, ERAP1 expression is inducible by IFNγ [10]. ERAP1 polymorphisms are associated with variable function and susceptibility to cancer, autoimmune disorders and infectious diseases, and its expression can be altered under pathological conditions [11]. Hence, various ERAP1 inhibitors have been developed to modulate antigen presentation, including GRWD5769, which is currently undergoing Phase I/II clinical trials in patients with advanced cancers [9, 12].

**Figure 2.**
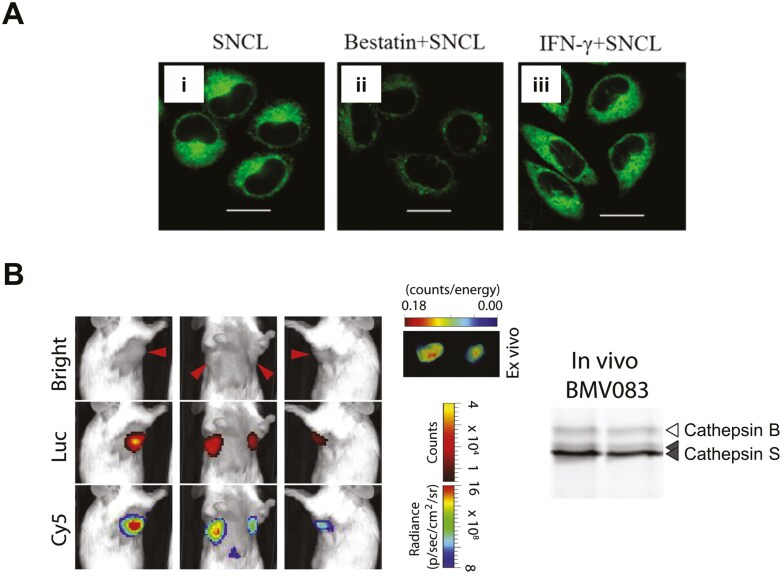
**Activity-based probes for visualizing the enzymology of antigen processing.** (A) Intracellular expression of ERAP1 was detectable using a two-photon fluorescent probe SNCL (i). Treatment with the aminopeptidase inhibitor bestatin resulted in lower uptake of SNCL (ii), whilst a higher uptake of the probe was detected in IFNγ-stimulated HeLa cells. (B) An activity-based probe BMV083 (Cy5 signal) for imaging cathepsin S activity showed localization to 4T1-luc-GFP tumours *in vivo*, whereby CD11b^+^ F4/80^hi^ MMR^+^ Gr-1^+^ cells (M2-type macrophages) were found to be the major cellular source of cathepsin S in the tumours. Fluorescent analysis of the SDS-PAGE gel of the tumour homogenates confirmed that cathepsin S was the major *in vivo* target of BMV083, with only minor labelling of cathepsin B. *Images reproduced with permission from* [14, 16].

Leucine-7-amino-5-methylcoumarin (Leu-Amc) is the only commercially available fluorogenic peptidyl substrate for quantifying leucine aminopeptidase activity, such as ERAP1. It is typically used in high-throughput screening of protease inhibitors to treat viral infections [13]. Enzymatic cleavage of Leu-Amc releases the AMC fluorophore, yielding a bright blue fluorescence. However, Leu-Amc is not specific for ERAP1, and its optical properties are not suitable for prolonged live cell imaging. Therefore, there is great interest in developing more specific imaging agents or tracers for detecting ERAP1 activity in the far red or NIR range [14, 15]. For instance, Xu et al. have developed a tracer (SNCL) containing a fluorophore (1,8-naphthalimide), a trigger moiety (*L*-leucine), and an ER targeting group (methyl sulfonamide) for probing ERAP1 in live cells and tumour tissues [14]. The tracer exhibited relatively low cytotoxicity in HeLa cells and high sensitivity towards ERAP1. Real-time imaging showed SNCL localizing immediately to the ER within 3 minutes of staining, and the fluorescent signal gradually increased in the cell over 30 minutes. A higher ERAP1 fluorescent signal was detected when cells were induced with IFNγ, whereas treatment with the aminopeptidase inhibitor bestatin led to signal reduction (**Fig. 2A**). ERAP1 activity was detectable by two-photon imaging on tumour sections at an imaging depth of 50–120 µm. This demonstrated its utility in future for live tissue imaging. However, the ubiquitous expression of ERAP1 is not ideal for whole-body imaging.

### Cathepsin S

Cathepsin S (CatS) is a cysteine protease crucial for Class II MHC antigen presentation. It facilitates proteolytic cleavage of the invariant chain to enable antigen loading onto MHC-II complexes [17]. CatS is mainly localized in the endo-lysosomal compartment, but also functions extracellularly in matrix remodelling. Unlike most cathepsins, CatS expression is restricted to antigen-presenting cells (APC) such as dendritic cells (DC), B cells, monocytes and macrophages, and non-professional APCs like intestinal epithelial cells. Therefore, CatS is abundant in lymphoid tissues. It is unique among cathepsins due to its wide pH range, being active at both neutral and acidic pH. CatS is overexpressed in several inflammatory disorders, e.g. chronic obstructive pulmonary disease and nociceptive pain, and is inducible by IFNγ. Tumour-associated macrophages (TAM) are the primary source of CatS in cancer [17]. Several CatS inhibitors are in clinical trials for treating autoimmune diseases, including RWJ-445380, which has completed a Phase II trial for rheumatoid arthritis patients (*NCT00425321*).

Various tracers have been developed for imaging CatS activity, including CatS-activatable dendrimers and activity-based tracers for NIRF imaging [16,18,19]. During macrophage recruitment to tumours, tumour microenvironment (TME) factors like interleukin-4, can induce CatS activity. Caglič et al. developed a “reverse design” ABP (AW-091) by converting the substrate-mimicking warheads of two CatS inhibitors into cleavable peptide bonds and attaching them to reporter groups [19]. AW-091 showed high selectivity for CatS *in vitro* and was effective in probing CatS activity in a mouse model of paw inflammation. It showed higher signal-to-background ratios at earlier imaging timepoints than the commercial pan-cathepsin imaging agent ProSense680. Fluorescent signal reduction was observed when mice were treated with the anti-inflammatory drug dexamethasone and the cathepsin inhibitor E-64.

Verdoes et al. developed a quenched ABP (BMV083) based on non-peptidic triazole inhibitors for imaging CatS activity in TAMs [16]. BMV083 demonstrated good cell permeability and labelling specificity *in vitro* for CatS at both neutral and acidic pH, primarily localizing to lysosomal compartments. It showed better stability *in vivo* compared to peptide-based imaging agents. Biodistribution studies in a 4T1 mouse breast cancer model (**Fig. 2B**) showed tracer localization to the tumours, as well as the liver and spleen, and the tracer reports primarily on CatS activity in M2-type TAMs.

## Antigen presentation

Antigen presentation involves the delivery of peptide-MHC (pMHC) to T cells to initiate a series of signalling cascades and cellular responses. The expression of several molecules including MHC, T cell receptors (TCR), T cell co-receptors, T cell co-stimulatory and co-inhibitory proteins at the immunological synapse, as well as the tissue localization and interactions between specific immune cell types influence T cell function during antigen recognition. Several tools have been generated for visualizing these components and will be useful for the development of imaging biomarkers to facilitate immunotherapy monitoring.

### Major histocompatibility complex

MHC, also known as the human leukocyte antigen (HLA) in humans, is a large family of glycoproteins crucial for antigen presentation to T cells. MHC molecules are subdivided into Class I, II, and III MHCs based on their structure and function. MHC-I molecules are expressed on all nucleated cells and are responsible for presenting intracellular peptides to CD8^+^ T cells [20]. MHC-I is constitutively expressed on cells and is inducible by IFNγ. Loss of MHC-I promotes immune evasion in cancer [21]. Low MHC-I expression has been found in immune-privileged sites, e.g. the central nervous system, and is upregulated in response to neuroinflammation [22]. Non-invasive imaging of MHC-I has not been reported in cancer, but a Cy5.5-conjugated high affinity peptide (H2BP) has been developed for imaging neuroinflammation in mice with cerebral ischaemic stroke [23]. H2BP is a peptide epitope (KALYNFATM) derived from the viral envelope glycoprotein gp33 of LCMV and could bind to mouse H-2K^b^ and H-2D^b^ MHC-I molecules. NIRF imaging showed significant uptake of H2BP in the ischaemic brain tissues at 4 hours post-injection compared to normal tissues and a control peptide tracer. *Ex vivo* biodistribution studies at 24 hours revealed high accumulation of H2BP in the kidneys indicating renal excretion as a possible route of clearance. Immunofluorescence staining confirmed MHC-I expression in ischaemic brain tissues, and colocalization with H2BP signals. Despite its ability to penetrate the blood brain barrier for imaging, this peptide-based tracer is prone to proteolysis and fast clearance. Thus, repeated tracer administration will be needed for later imaging timepoints that could impede clinical translation. Furthermore, many of the described human HLA class I epitopes of tumour antigens, e.g. NY-ESO-1 and Melan-A/MART-1 have low affinity as predicted by the Immune Epitope Database Analysis Resource [24]. This can influence pMHC binding and image signal retention in tissues, thus the use of a peptide-based tracer for imaging MHC-I needs to be carefully considered. In addition, the physicochemical properties and MHC binding affinity of short peptides may be affected by the addition of fluorophores. To improve the specificity in detecting MHC-I presentation of tumour epitopes in cancer, one could consider modifying TCR mimic antibodies used in cancer immunotherapy for imaging applications [25].

MHC-II molecules are usually expressed on APCs, but can be induced on epithelial and cancer cells under inflammatory conditions [20]. They present exogenous antigens taken up by APCs to CD4^+^ T helper cells. Aberrant MHC-II expression has been reported in malignancies like diffuse large B cell lymphoma (DLBCL) and oesophageal cancer [26, 27]. Loss of HLA-DR (a human MHC-II) is associated with less CD4^+^ T helper cell infiltration and poorer survival in DLBCL [26]. High MHC-II expression correlates with an inflamed TME and predicts response to anti-PD-1/PD-L1 therapy in melanoma and NSCLC [28, 29]. HLA-DRB4 is the predominant genotype among NSCLC patients experiencing endocrine immune-related adverse events (irAE) [30]. These findings suggest that MHC-II is a valuable biomarker for evaluating immune response in cancer.

**Figure 3.**
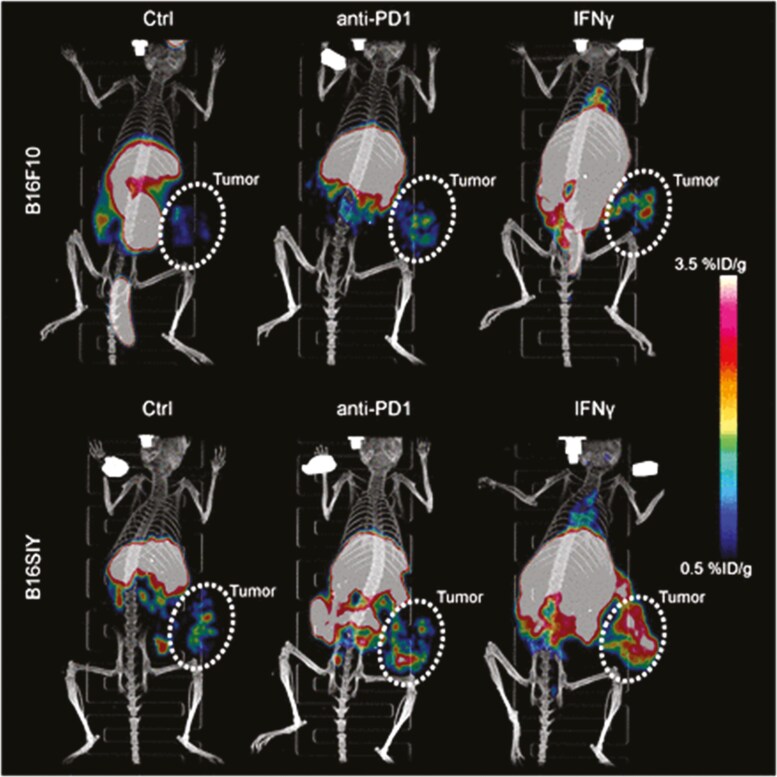
**Imaging major histocompatibility complex during cancer immunotherapy.** Immuno-PET imaging of MHC-II expression in mouse melanoma models using ^64^Cu-DOTA-MHCII during PD-1 immunotherapy and IFNγ stimulation. %ID/g in the scale bar represents the percentage injected radiotracer dose per gram of tissue. *Image reproduced with permission from* [31].

Yang et al. developed a Copper-64 radiolabelled antibody (^64^Cu-DOTA-MHCII) for positron emission tomography (PET) imaging of MHC-II expression in mouse melanoma models [31]. Higher uptake of ^64^Cu-DOTA-MHCII was observed in mice with more immunogenic B16SIY tumours compared to B16F10. Tumour accumulation of the radiotracer was higher when mice were treated with anti-PD-1, and further increased with IFNγ stimulation (Fig. 3). These correlated with MHC-II expression on western blot. High radiotracer accumulation was detected in the liver and spleen due to the presence of MHC-II expressing cells. Immunohistochemistry confirmed MHC-II expression was mainly in CD45-negative cancer cells. However, the use of full-length antibodies for *in vivo* imaging has limitations due to their considerable size (~150 kDa) which results in long circulatory half-life and poor tissue penetration. Therefore, smaller antibody fragments have been developed for imaging [32]. One example is the use of camelid nanobodies (~15 kDa) for probing HLA-DR in a humanized mouse model of graft-versus-host-disease (GvHD) [33]. Nanobodies (VHHs) consist of single monovalent antibody variable domains that retain antigen-binding capabilities, improved stability, short circulatory half-life and increased tissue penetration. *Ex vivo* two-photon imaging of the spleen, lymph nodes (LN) and thymus from humanized mice administered with a fluorescent version of the tracer showed the germinal centre B cells, thymic APCs and thymic epithelial cells were stained positively with the tracer. Non-invasive, whole-body imaging with Immuno-PET revealed high uptake of the radiotracer (^64^Cu-VHH4) in the spleen and bone marrow, indicating the presence of HLA-DR-positive cells. Non-specific ^64^Cu-VHH4 accumulation was observed in common sites of VHH clearance (kidney and bladder). An intense PET signal was detected in the inflamed livers of mice with stage 3 GvHD. This corresponded to higher human T cell infiltration and increased HLA-DR expression on activated T cells seen on flow cytometry. However, no PET signal was detected in other organs that could potentially be affected in GvHD like the gut, possibly due to the lower density of HLA-DR-positive cells or scavenging of labelled nanobodies by organs with more HLA-DR-positive cells.

### T cell receptor

The TCR is a member of the immunoglobulin superfamily important for recognizing specific pMHC. Each TCR consists of two polypeptide chains linked by disulfide bonds. In humans, 95% of the T cells express TCR composed of α and β chains, while 5% express γδ TCRs. Each chain contains a variable domain with three complementarity-determining regions for antigen recognition, a constant domain, a transmembrane region, and a short cytoplasmic tail. The TCR is expressed along with the CD3 complex to facilitate signal transduction following TCR-pMHC binding [34].

**Figure 4.**
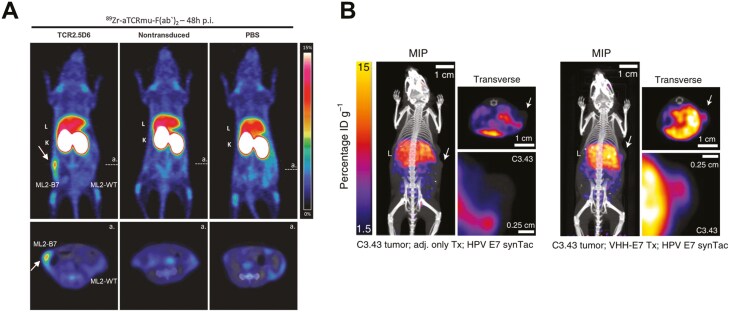
**Imaging the T cell receptor.** (A) Immuno-PET imaging enables whole-body detection of biomarker expression. Imaging murine TCRβ constant domain using ^89^Zr-aTCRmu-F(ab’)_2_ following adoptive transfer of TCR2.5D6-transduced T_CM_ into NSG mice bearing bilateral ML2-B7 and ML2-WT tumours. Arrow indicates radiotracer accumulation in ML2-B7 tumours of mice infused with TCR2.5D6-transduced T_CM_. L: liver; K: kidneys [37]. (B) Immuno-PET imaging of mouse HPV E7-specific TCR using ^64^Cu-radiolabelled HPV E7 synTacs in mice bearing HPV16 E7-positive C3.43 tumours. Radiotracer uptake in mice immunized with VHH_CD11b_-E7 compared to adjuvant only. White arrow indicates position of C3.43 tumour. L: liver. %ID g^-1^ in the scale bar represents the percentage injected radiotracer dose per gram of tissue. Images were adapted from [38]. *All images reproduced with permission from* [36–38].

T cell activation upon cognate antigen engagement is dependent on the dynamics and spatial distribution of TCRs and downstream signalling molecules. These nanoscale subdiffractional organization of TCRs are best visualized with super-resolution microscopy and have been widely studied in literature [35, 36]. At the whole-body level, a Zirconium-89 labelled antibody fragment, ^89^Zr-aTCRmu-F(ab’)_2_ has been developed for imaging the murine TCRβ constant domain [37]. This radiotracer tracked the whole-body distribution of human central memory CD8^+^ T cells (T_CM_) transduced with TCR2.5D6 which contains murine constant domains in the α and β chains and is specific for the myeloperoxidase-derived peptide (MPO_5_) presented on HLA-B7 in acute myeloid leukaemia (AML). *In vitro* validation of aTCRmu-F(ab’)_2_ showed no significant impairment on the cell viability and IFNγ secretion of TCR2.5D6-transduced T_CM_ compared to the full-length antibody aTCRmu-IgG. Immuno-PET imaging conducted at 3 days after adoptive transfer of TCR2.5D6-transduced T_CM_ into NSG mice bearing bilateral ML2-B7 (transduced with HLA-B7) and ML2-WT human AML tumours showed a 4-fold higher uptake of ^89^Zr-aTCRmu-F(ab’)_2_ in ML2-B7 tumours (Fig. 4A). Mice infused with non-transduced T_CM_ or PBS showed no difference in radiotracer accumulation between both tumours. Higher splenic and lung uptake was detected in mice with TCR2.5D6-transduced T_CM_. However, the antibody used for tracer development is of hamster origin and a transgenic TCR with murine components may lead to immunogenicity issues and impede clinical translation. Future studies may consider humanization of aTCRmu-F(ab’)_2_ and reducing the murine gene segments in the TCR construct.

On the other hand, Fc-based pMHC dimers called synTac (synapse for T cell activation) have been developed for imaging specific TCRs [38, 39]. synTacs are produced in mammalian cell expression systems and have reduced immunogenicity. The pMHC module is covalently bound and the peptide is associated with the MHC in a stable, non-exchangeable manner. However, the peptide sequence can be altered to detect different T cell specificities. The Fc region of synTacs was modified with a sortase recognition motif (LPETG) for site-specific conjugation of (Gly)_3_-radiometal chelators, facilitating whole-body imaging. *In vitro* validation of synTacs targeting the HPV E7 peptide showed specificity, CD8^+^ T cell activation and IFNγ secretion in response to the peptide. Immuno-PET imaging using ^64^Cu-radiolabelled HPV E7 synTacs demonstrated higher radiotracer accumulation in C3.43 HPV16 E7-positive tumours of mice immunized with HPV E7 peptide conjugated to anti-CD11b VHH (VHH_CD11b_-E7) compared to the adjuvant-only group (**Fig. 4B**). However, high radiotracer accumulation in the liver was detected, likely due to the molecular size of synTacs (~163 kDa) where large-sized proteins are usually cleared via the hepatobiliary route, sequestration of ^64^Cu in the liver due to copper metabolism, or the presence of antigen-specific T cells in the liver and scavenging of the radiotracer.

### T cell co-receptors

The T cell co-receptors CD3, CD4, and CD8 play important roles in TCR signalling and defining T cell lineages. CD3 is a pan-T cell marker, comprising of a protein complex with a CD3γ chain, a CD3δ chain and two CD3ε chains. These chains associate with the TCR and ζ-chains to generate T cell activation signals. The intracellular domain of CD3 contain immunoreceptor tyrosine-based activation motifs that can be phosphorylated by the tyrosine kinase Lck, allowing binding to ZAP-70 and triggering the TCR signalling cascade [34]. Because CD3 is essential for T cell activation, monoclonal antibodies (mAb) have been developed as immunosuppressants for treating autoimmune diseases and transplant rejection. In addition, CD3-based bispecific antibodies serve as T cell engagers to redirect T cells towards tumours for killing. When used in sub-therapeutic microdoses, mAbs targeting CD3 can be applied to whole-body imaging of T cell infiltration in tissues.

**Figure 5.**
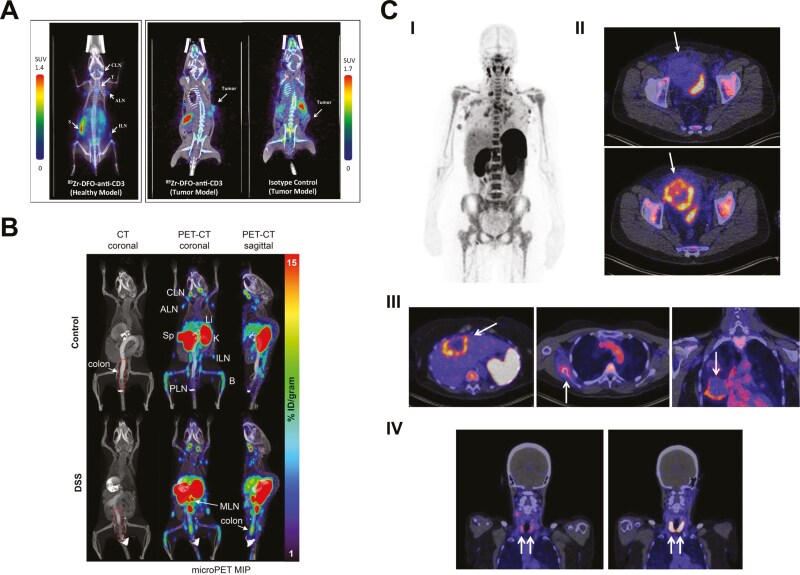
**Whole-body imaging of T cell co-receptors in patients and preclinical models.** (A) Immuno-PET imaging of CD3 in syngeneic mouse models of bladder cancer using ^89^Zr-DFO-anti-CD3 compared to radiolabelled isotype control IgG. SUV: standard uptake value of radiotracer; White arrow indicates position of tumour. CLN: cervical LN; ALN: axillary LN; ILN: inguinal LN; T: thymus; S: spleen [40]. (B) Immuno-PET imaging of CD4 in dextran sulfate sodium (DSS) mouse model of colitis using ^89^Zr-malDFO-GK1.5 cDb. CLN: cervical LN; ALN: axillary LN; ILN: inguinal LN; PLN: popliteal LN; MLN: mesenteric LN; Sp: spleen; Li: liver; K: kidneys; B: bone [41]. (C) Clinical trial on imaging human CD8 in cancer patients before and after immune checkpoint blockade using ^89^ZED88082A (*NCT04029181*). I: Maximum intensity projection image of whole-body ^89^ZED88082A uptake on day 2 following tracer injection. II: Radiotracer uptake in dMMR urothelial tumour before (top image) and after (bottom image) treatment. III: High rim uptake of radiotracer in liver metastases of a patient with dMMR colorectal cancer (left image), bone lesion in a patient with squamous cell vulvar cancer (middle image) and lung metastatic lesion in a patient with cervical cancer (right image). IV: High radiotracer accumulation in the thyroids of a patient with Hashimoto’s thyroiditis before treatment (left image) that further increase in uptake after a flare-up during immunotherapy (right image). Images were adapted from [42]. *All images reproduced with permission from* [40–42].

Vera et al. have generated ^89^Zr-DFO-anti-CD3 for detecting CD3 expression in syngeneic mouse models of bladder cancer [40]. Immuno-PET imaging revealed preferential accumulation of the radiotracer in the spleen, LNs, thymus and bone marrow. An 11.5-fold increase in tumour-to-blood signal was observed in tumours of mice injected with ^89^Zr-DFO-anti-CD3 compared to the isotype control IgG (Fig. 5A). A similar radiotracer was used for probing CD3 expression in the CT26 mouse colorectal cancer model following anti-CTLA-4 therapy [43]. Radiotracer uptake was significantly higher in the responding tumours than in non-responding ones, despite no significant difference in tumour volume. This demonstrated the feasibility of using ^89^Zr-DFO-anti-CD3 for early detection of immunotherapy response. However, high radiotracer uptake was observed in the liver due to hepatobiliary clearance of large-sized antibodies. The presence of intact Fc regions could potentially lead to antibody-dependent cell-mediated cytotoxicity (ADCC) and depletion of the target cells. Imaging T cells using full-length antibodies is limited, especially for lesions in the liver and surrounding abdominal regions. Future studies may consider using CD3 antibody fragments for *in vivo* imaging.

The use of radiolabelled antibody fragments to improve tracer pharmacokinetics while retaining antigen specificity and binding affinity has been explored for imaging CD4 and CD8 [41, 42, 44–47]. CD4 is a transmembrane glycoprotein expressed on MHC-II restricted T cells. It is present to a lesser extent on some monocytes, macrophages, certain DC populations and Langerhans cells, some B cells and brain microglial cells. Changes in CD4^+^ T cell infiltration into tissues has been associated with various chronic inflammatory disorders, including colitis, HIV and cancer. Wu and colleagues developed a radiolabelled anti-mouse CD4 cys-diabody (^89^Zr-malDFO-GK1.5 cDb) for imaging CD4^+^ T cells in a mouse model of colitis [41]. Cys-diabodies are small (~55 kDa) and lack Fc regions, enabling rapid clearance and a high target-to-background signal ratio for imaging. Cysteine residues are engineered into the C-terminal region for site-specific conjugation of metal-chelators [48].

Immuno-PET imaging showed normal tracer accumulation in the spleen and LNs of both diseased and normal mice. Radiotracer uptake was significantly higher in the distal colon and mesenteric LNs of diseased mice compared to controls (**Fig. 5B**). This was confirmed by immunohistochemistry and flow cytometry, which showed greater infiltration of CD4^+^ T cells in these tissues. This suggested potential application of CD4 Immuno-PET imaging in monitoring colitis induced by immune checkpoint inhibitors.

CD8 is a transmembrane glycoprotein predominantly expressed on cytotoxic T cells. It can also be found on NK cells, DCs and cortical thymocytes. It is present on T cell surface as dimers in two isoforms, CD8αα or CD8αβ [34]. Several antibody fragment-based CD8 tracers have been developed for Immuno-PET imaging [41, 42, 44–47]. These include a CD8-targeted minibody (^89^Zr-Df-Crefnirlimab) by ImaginAb, which is actively tested in clinical trials globally for several applications including cancer, COVID-19 infection and autoimmune disorders [44–46]. A radiotracer (^89^ZED88082A) based on a 100 kDa “one-armed” CD8 antibody was generated by Genentech for monitoring CD8 status during cancer immunotherapy [42, 47]. This monovalent antibody format was chosen to avoid cross-linking CD8 on the cell surface and modulating TCR signalling upon tracer binding. The Fcγ binding is silenced to prevent ADCC and depletion of CD8^+^ T cells, while FcRn binding is retained to prolong circulatory half-life and promote tissue penetration and CD8 exposure to the tracer.

SPR analysis showed that the antibody binds human CD8 with nanomolar affinity. Flow cytometric analysis demonstrated selectivity in antibody binding to human CD8^+^ T cells, but not CD4^+^ T cells in mixed PBMCs [47]. Immuno-PET imaging of cancer patients before and after immune checkpoint blockade (*NCT04029181*) revealed ^89^ZED88082A uptake in normal lymphoid tissues, as well as the liver and kidneys which are the main clearance organs (**Fig. 5C**) [42]. No tracer-related side effect was reported. Heterogeneity in radiotracer accumulation was detected in tumour lesions within and between patients. A pronounced rim uptake of the radiotracer was noted in tumours with DNA mismatch repair (dMMR) deficiency, and higher radiotracer accumulation was associated with longer overall survival. Higher uptake was also observed in tumours with inflamed or stromal phenotype compared to immune desert phenotype. This was confirmed by autoradiography and immunohistochemistry, which showed tissue radioactivity in areas with CD8 expression. Interestingly, high radiotracer accumulation was detected in the thyroids of a patient with Hashimoto’s thyroiditis that further increased in uptake after a flare-up following treatment with immune checkpoint inhibitors. However, no difference in radiotracer uptake was detected in other patients experiencing irAE of grade ≥ 3 in this small prospective trial.

### T cell function

Whole-body imaging complements conventional immunological assays in evaluating T cell function following antigen recognition. This approach provides a global view of immune processes and enables the non-invasive monitoring of disease progression and response to immunotherapy. Numerous radiotracers targeting co-stimulatory molecules, immune checkpoints, T cell activation markers, and cytokines have been developed for Immuno-PET imaging [49–64]. The expression of these T cell functional markers can influence the quality of TCR signalling following cognate antigen engagement, as well as T cell’s ability to sustain its activity in eradicating cancer. Many of these tracers are already undergoing clinical testing, as summarized in Table 1.

**Table 1.** Radiopharmaceuticals for Immuno-PET imaging of T cell function following antigen recognition

Target	Radiopharmaceutical	Application	Stage of development	References
**T Cell Co-stimulation**				
ICOS	^89^Zr-DFO-ICOS mAb	Imaging ICOS expression following treatment with STING agonist and CAR T cell therapy	Preclinical	[49, 50]
OX40	^64^Cu-DOTA-AbOX40 ^89^Zr-DFO-OX40 mAb	Imaging OX40 expression following treatment with cancer vaccine	Preclinical	[51, 52]
**Immune Checkpoints**				
CTLA-4	^89^Zr-ipilimumab	Imaging tumour uptake and biodistribution of ipilimumab	Clinical *NCT03313323*	[53]
PD-1	^89^Zr-nivolumab	Imaging PD-1 expression before nivolumab treatment	Clinical *EudraCT 2015-004760-11*	[54]
PD-L1	^18^F-BMS-986192	Imaging PD-L1 expression before nivolumab treatment	Clinical *EudraCT 2015-004760-11*	[54]
LAG-3	^89^Zr-BI 754111	Imaging tumour uptake and biodistribution of BI 754111	Clinical *NCT03780725*	[55]
Tim-3	^64^Cu-NOTA-RMT3-23	Imaging Tim-3 expression following radiotherapy	Preclinical	[56]
TIGIT	^89^Zr-TIGITmAb	Imaging TIGIT expression on tumour-infiltrating lymphocytes	Preclinical	[57]
**T Cell Activation**				
CD69	^89^Zr-DFO-H1.2F3 ^68^Ga-DOTA-Z_CAM241_	Imaging CD69 expression following immune checkpoint blockade Imaging CD69 expression in inflammatory arthritis	Preclinical	[58, 59]
CD25 (IL2Rα)	^18^F-FB-IL2	Imaging IL2Rα expression before and during treatment with immune checkpoint inhibitors	Clinical	[65]
GzmB	^68^Ga-NOTA-GZP ^68^Ga-grazytracer	Imaging GzmB expression following immune checkpoint blockade (CD25) Imaging GzmB expression following immune checkpoint blockade or adoptive T cell transfer	Preclinical Clinical *NCT05000372*	[60] [61]
**Cytokines**				
IFNγ	^89^Zr-anti-IFNγ	Imaging IFNγ expression following treatment with cancer vaccine	Preclinical	[62]
TNFα	^89^Zr-DFO-CZP ^89^Zr-DFO-infliximab	Imaging TNFα expression in rheumatoid arthritis Imaging TNFα expression in colitis	Preclinical	[63, 64]

*Cytotoxic T lymphocyte antigen 4; PD-1: Programmed cell death protein 1; PD-L1: Programmed death ligand 1; LAG-3: Lymphocyte activation gene 3; Tim-3: T cell immunoglobulin- and mucin-domain-containing 3; TIGIT: T cell immunoreceptor with Ig and ITIM domains; CD25: Interleukin-2 receptor α chain; CD69: Type II C-lectin membrane receptor; GzmB: Granzyme B; IFNγ: Interferon-gamma; TNFα: Tumour necrosis alpha; Radionuclides—* ^*18*^ *F: Fluorine-18;* ^*64*^ *Cu: Copper-64;* ^*68*^ *Ga: Gallium-68;* ^*89*^ *Zr: Zirconium-89; Metal chelators—DFO: Deferoxamine; DOTA: 1,4,7,10-terraazacyclododecane-1,4,7,10-tetraacetic acid; NOTA: 1,4,7-triazacyclononane-1,4,7-triacetic acid. List updated as of August 2024.*

Notably, a radiotracer (^89^Zr-DFO-OX40 mAb) has been developed by the Gambhir group for imaging OX40 expression following vaccination [52]. OX40 is a relevant biomarker for assessing the efficiency of antigen presentation. Unlike other co-stimulatory molecules such as CD28, OX40 is not constitutively expressed on resting naïve and memory T cells; instead, it is induced on activated T cells for 24 to 72 hours following TCR engagement. Its expression is restricted to activated antigen-specific T cells, unlike other activation markers like CD25 and CD44, which are associated with various cell types. OX40 binding to OX40L activates immune pathways that regulate T cell activation, differentiation, proliferation, and survival [66].

Immuno-PET imaging in an orthotopic mouse glioma model showed significant uptake of ^89^Zr-DFO-OX40 mAb in the spleen, tumour-draining LNs (cervical) and LNs near the vaccinated site (axillary) of mice treated with a cocktail of CpG-oligonucleotides, tumour lysates, and OX40 mAbs compared to controls. Flow cytometry confirmed significantly higher infiltration of OX40^+^ CD4^+^ T cells in these tissues. However, brain tumour uptake was moderately higher in the controls than in vaccinated mice, despite no significant difference in OX40^+^ CD4^+^ T cell infiltration. This discrepancy may arise from enhanced permeability and retention effects and vascular leakiness in the larger brain tumours of the control mice, leading to non-specific tracer accumulation.

Chronic T cell stimulation during antigen presentation can lead to exhaustion. Various biologics have been generated to block immune checkpoint proteins, such as CTLA-4, PD-1, and LAG-3, with several of these agents now routinely used in clinical practice or undergoing trials. Concurrently, imaging biomarkers are being developed as companion diagnostics alongside with these immunotherapeutic advancements. These imaging tools can potentially aid evaluation of T cell exhaustion during antigen presentation. For instance, radiotracers for imaging PD-1 (^89^Zr-Nivolumab) and PD-L1 (^18^F-BMS-986192) expression in NSCLC patients prior to treatment with nivolumab have been developed [54]. Tumour uptake of ^89^Zr-Nivolumab correlated with PD-1 expression on tumour-infiltrating immune cells on biopsy, while ^18^F-BMS-986192 uptake was associated with tumour PD-L1 expression. Radiotracer uptake was heterogenous among patients and across different tumour lesions within the same patient. Notably, higher pre-treatment tumour uptake of ^89^Zr-Nivolumab correlated with better clinical outcomes with nivolumab. Interestingly, some tumours exhibited low PD-L1 expression on biopsy despite relatively high ^18^F-BMS-986192 uptake.

Similarly, in another study on imaging LAG-3 expression (^89^Zr-BI 754111) in NSCLC and head and neck cancer patients with progressive disease following PD-1 therapy, tumour heterogeneity in tracer uptake, and discrepancies between high tracer uptake and low LAG-3 expression on tumour biopsy were observed [55]. This may result from the inherent tumour heterogeneity in PD-L1 and LAG-3 expression, which is often difficult to capture in small biopsy specimens [67]. Therefore, non-invasive imaging can serve as a complementary biomarker for patient stratification and predicting immunotherapy response. Furthermore, the pharmacokinetics and whole-body distribution of these biologics can be assessed in living patients to determine whether therapeutic proteins reach target sites and identify any off-target effects.

T cell activation and cytokine release following antigen stimulation is a time-limited process. Immunological activities occurring *in vivo* may not be fully captured using processed tissues *in vitro* with conventional methods like flow cytometry and immunohistochemistry. For instance, CD69 is an early marker of T cell activation, expressed within 4 hours after TCR engagement, peaking at 24 hours, and declining immediately after 120 hours; CD25, in contrast, is expressed later at 8 hours and peaks only after 96 hours [68]. The kinetics and patterns of granzyme B (GzmB), IFNγ, and TNFα expression also depend on the conditions under which T cells are activated [69–71]. Thus, non-invasive imaging using Immuno-PET provides additional insights on the spatiotemporal dynamics of T cell activation in living organisms and advances our understanding of T cell biology during immunotherapy.

Several radiotracers have been developed for imaging T cell activation and cytokine release *in vivo* [58–65]. For example, a CD69 affibody (^68^Ga-DOTA-Z_CAM241_) was created for early detection of inflammatory arthritis [59]. The radiotracer showed increasing uptake in joints on days 3, 7, and 12 after inflammatory arthritis induction in a KRN adoptive T cell transfer model, with changes detectable even at day 3, prior to any clinical signs of joint inflammation. A peptidomimetic (^68^Ga-grazytracer) has also been developed for imaging GzmB activity during immune checkpoint blockade [61]. The tracer design is based on a GzmB-targeting tetrapeptide aldehyde (Ile-Glu-Pro-Asp) identified from combinatorial library screening. Unlike conventional linear peptide tracers, ^68^Ga-grazytracer was synthesized with a rigid tricyclic scaffold for improved *in vivo* stability and a non-aldehyde 1,2,3-triazole moiety to enhance selectivity as a GzmB inhibitor. The tracer showed nanomolar affinity for GzmB, good binding specificity for both mouse and human GzmB and metabolic stability *in vivo*.

Uptake of ^68^Ga-grazytracer was predictive of immunotherapy response in mouse colorectal and lung cancer models treated with immune checkpoint inhibitors, and imaging was able to distinguish pseudoprogression from true tumour progression. A pilot study on five patients indicated that those patients with positive ^68^Ga-grazytracer uptake exhibited better treatment response whereas those with negative uptake showed poorer response. Lastly, elevated IFNγ levels were detected using a mAb-based tracer targeting IFNγ (^89^Zr-anti-IFNγ) in neu+ mouse breast tumours following treatment with HER2/neu DNA vaccines [62]. This corresponded with increased IFNγ, CD3 and CD8 expression observed on qPCR. Conversely, in a model of passive immunotherapy (neu TAA-specific mAb injection), CD8^+^ T cells infiltrating the tumours were found to be exhausted (PD-1^+^) with poor IFNγ production, a phenomenon also captured through imaging.

### Tertiary lymphoid structures

Tertiary lymphoid structures (TLS) are highly organized clusters of lymphocytes and APCs that form in non-lymphoid tissues during chronic inflammation. They serve as local hubs for antigen presentation, humoral response and adaptive immune activation in the periphery. TLS share structural and functional characteristics as secondary lymphoid organs, including distinct T and B cell zones, marginal zones of activated DCs and macrophages, reticular fibroblast networks, and high endothelial venules. However, TLS lack capsules and afferent lymphatic vessels [72]. These transient structures can be triggered by immunization or infection, and often resolve after antigen clearance [73]. In cancer, the presence of intratumoural TLS, along with B cell maturation and antibody production, is associated with response to immunotherapy [74, 75]. Additionally, the location and density of TLS predict overall survival in patients with metastatic cancers [76, 77]. Imaging methods that elucidate the spatial landscape of TLS will enhance disease and treatment monitoring and improve our understanding of the mechanisms involved in antigen presentation.

Traditionally, H&E and immunohistochemical staining of CD20 and CD23 for B cells and follicular DCs are used to identify TLS in tissue sections. Advancements in imaging and sequencing technologies now enable the quantification and mapping of gene and protein expression of multiple cell types in relation to their tissue location and cellular neighbourhood [78]. For instance, highly multiplex imaging techniques such as imaging mass cytometry (IMC) and co-detection by indexing (CODEX) can simultaneously detect over 40 different protein targets at subcellular resolution in tissues using metal-tagged or fluorescent DNA-barcoded antibodies [79, 80]. Spatial transcriptomics, based on *in situ* capture technology and spatially barcoded oligonucleotides like 10X Visium, enables whole transcriptome analysis at the single cell level. Additionally, cyclic fluorescent *in situ* hybridization methods, such as the CosMx Spatial Molecular Imager, supports simultaneous imaging and quantification of up to 1,000 RNA and 64 protein targets at subcellular resolution [81]. These platforms provide further insights on various cell types, functional states and their spatial organization within tissues. For instance, IMC has been used to characterize TLS morphology in tumours of patients with hepatocellular carcinoma [79]. Matured TLS were present in the viable tumours of patients treated with neoadjuvant immunotherapy but absent in the untreated patients. In regions of tumour regression, an involuted (resolving) TLS morphology was observed, with on-going dissolution of the B cell follicle, persistence of a T cell zone enriched for antigen presentation and T cell-DC interactions, increased expression of T cell memory markers, and expansion of cytotoxic and tissue-resident memory clonotypes. Another study using spatial transcriptomics revealed TLS as sites of *in situ* B cell maturation into plasma cells in renal cell carcinoma [75]. Spatial B cell receptor profiling demonstrated clonal diversification, selection and expansion within TLS, as well as the presence of fully mature clonotypes at a distance. This depth of analysis was possible as the Visium platform is based on RNA sequencing of barcoded transcripts.

In the realm of *in vivo* imaging, several reporter mice have been developed for intravital microscopy to study lymphoid architecture, antigen presentation and specific immune cell behaviour. These approaches could be repurposed for live imaging of the TLS microenvironment. For example, Prox1-GFP mice visualize lymphatic vessels [82], OT1-GFP mice are classic models for studying antigen-specific CD8^+^ T cell trafficking and interactions with APCs [83], and XCR1-Venus mice selectively label the cDC1 subtype that will be useful for imaging cross-presenting DCs in tissues [84]. B^RedDi^ mice enables tracking and selective depletion of B cells, as this transgenic strain expresses RFP along with the diphtheria toxin receptor under a B cell-specific *mb-1* promoter [85]. Although intravital microscopy allows for direct visualization of single-cell behaviour and cell-cell interactions in tissues, it is limited by field-of-view and light penetration depth. Terminal imaging of exposed tissues is invasive, and determining the right timepoint for imaging TLS—given their transient nature—can be challenging [86]. Window chamber models may be more useful for longitudinal tracking of TLS formation and cellular alterations during treatment [87].

As TLS are composed of complex cellular clusters, there is currently no defined biomarker for whole-body imaging. However, tracers are available for detecting major components of the TLS. For instance, Technetium-99m labelled Albumin Nanocoll consists of colloidal nanoparticles that can be taken up by phagocytes. It has been widely used for sentinel LN localization in cancer patients and has successfully detected pancreatic TLS in lupus-prone mice [88]. CD20^+^ B cells can be visualized with Immuno-PET in the tumours, spleen and draining LNs of human CD20 transgenic mice implanted with 4T1 tumours using the antibody fragment-based tracer ^89^Zr-CD20cMb [89]. Treatment with intratumoural CpG-oligonucleotides resulted in tumour growth inhibition and increased tracer accumulation in tumours and lymphoid tissues, indicating systemic immune activation that was further confirmed through immunohistochemistry. In humans, ^89^Zr-rituximab uptake before starting rituximab (anti-CD20) therapy was associated with treatment response in rheumatoid arthritis. A positive correlation was found between LN uptake of the tracer and immunohistochemistry [90]. Lastly, DC migration and localization within TLS can be tracked using PET and magnetic resonance imaging (MRI) [91, 92]. MRI provides superior spatial resolution for soft tissue imaging compared to other whole-body imaging modalities and does not involve ionizing radiation. DCs can potentially be modified with MRI reporter genes, or pre-labelled *ex vivo* with superparamagnetic iron oxide nanoparticles or Fluorine-19 perfluorocarbons before infusion into mice or patients for whole-body tracking of DC distribution, migration to and within lymphoid structures [92, 93].

## Conclusion

Antigen processing and presentation are essential processes of the adaptive immune system that significantly influence the outcomes of cancer immunotherapy. Advancements in imaging technologies—such as super-resolution microscopy, spatial transcriptomics, and whole-body imaging techniques like Immuno-PET and MRI—have enabled multi-scale visualization of the cellular and molecular processes involved in these mechanisms. Imaging has become an invaluable and complementary tool in basic immunology and translational medicine studies, providing new perspectives that enhance our understanding of tumour immunobiology, disease progression, early detection of treatment response, and occurrence of irAEs. The non-invasive nature of whole-body imaging further facilitates *in vivo* screening of immunotherapeutic drugs that could potentially “fast-track” pharmaceutical development while minimizing off-target side effects. The clinical translation of these diagnostic and prognostic biomarkers is crucial for tailoring treatments to individual responses and improving monitoring strategies. Future research should focus on integrating these imaging modalities with therapeutic approaches to optimize immunotherapy and further elucidate the complexities of immune response in cancer. Ultimately, these efforts aim to enhance patient outcomes, improve survival rates, and elevate the quality of life for individuals affected by cancer.
